# Telemonitoring Adoption Among Older Adults: Sequential Mixed Methods Study of Message Framing and Structural Influence

**DOI:** 10.2196/89817

**Published:** 2026-10-27

**Authors:** Dandan Wang, Huanyu Bao, Amber Pei Tze Qua, Gaelmaine Yu Heng Hoong, Pei Shan He, Ern Sze Maegan Ong, Edmund Wei Jian Lee

**Affiliations:** 1 Department of Media and Communication, City University of Hong Kong, 5F, Run Run Shaw Creative Media Centre, Hong Kong, China (Hong Kong), +852 34428633; 2 Wee Kim Wee School of Communication and Information, Nanyang Technological University, Singapore, Singapore; 3 Institute of Digital Medicine, City University of Hong Kong, Hong Kong, China (Hong Kong)

**Keywords:** telemonitoring, older adults, health communication, message framing, digital inequality, aging in place

## Abstract

**Background:**

Telemonitoring offers solutions for remote health management among older adults; yet, acceptance varies based on individual perceptions and presentation strategies.

**Objective:**

This 2-phased sequential mixed methods study examines the role of message framing on telemonitoring adoption among low-income older adults.

**Methods:**

Phase 1 involved in-depth interviews with 17 older adults, identifying key facilitators (accessibility, affordability, and health benefits) and barriers (preference for in-person consultations, technological limitations, and information processing difficulties) of telemonitoring. Building on these insights, phase 2 used an experimental study with 120 participants assessing message framing effects (episodic vs thematic) and modalities (video vs photo-text) on telemonitoring adoption.

**Results:**

Episodic framing did not produce a significant main effect on attitudes toward telemonitoring; however, its effect was moderated by external locus of control (LOC), with episodic framing associated with more positive attitudes among participants lower in external LOC, and thematic framing more favorable among those higher in external LOC.

**Conclusions:**

Convenience, cost, and health benefits emerged as key adoption drivers, while traditional consultation preferences and cognitive processing challenges posed barriers. Theoretical and practical implications were discussed.

## Introduction

### Background

Telemonitoring uses electronic technology to remotely track patients’ vital signs and health parameters [1]. By enabling continuous monitoring, telemonitoring facilitates early detection of health changes and supports “aging in place,” enabling older adults to live in their communities safely and independently [2,3]. However, telemonitoring services are not equitably accessible due to communication inequalities (disparities in access to and use of ITs), particularly among socioeconomically vulnerable groups [4]. Older adults face greater obstacles in receiving virtual care due to limited use of digital health technologies, insufficient technological resources, and lack of motivation [5]. Those with limited financial resources may lack access to necessary devices [6].

To address these gaps, we conducted a sequential mixed methods study grounded in the Structural Influence Model (SIM) and message framing theory. The SIM posits that health behaviors are shaped by social, economic, and environmental contexts [4], while message framing suggests that information presentation influences attitudes and decisions regarding health behaviors [7,8]. Phase 1 used qualitative methods to examine motivations and barriers for telemonitoring adoption among socioeconomically disadvantaged older adults. Phase 2 evaluated the effectiveness of different message framing approaches (episodic vs thematic) and modalities (video vs photo-text) to evoke motivations identified in phase 1 and therefore enhance telemonitoring acceptance, with attention to how health locus of control (LOC) moderates these effects. By investigating both the factors influencing telemonitoring adoption and testing targeted communication strategies, our research aims to contribute to the development of more inclusive and effective telemonitoring interventions that can help bridge the digital divide and improve health outcomes for socioeconomically disadvantaged older adults. This work responds to calls within social gerontology for research that addresses the intersection of technological innovation, social inequality, and the diverse lived experiences of aging [9,10], ultimately supporting the creation of more equitable models of digital health that enhance older adults’ agency and quality of life in their preferred living environments.

### Theoretical Framework

#### Structural Influence Model and Phase 1: Exploring Telemonitoring Adoption Among Older Adults

The SIM provides a theoretical framework for understanding how social determinants interact with communication processes to influence health behaviors and outcomes [11]. This model posits that communication outcomes, including exposure, usage, attention, seeking, and processing of health information, are interlinked with social determinants to influence health outcomes (Figure 1). According to SIM, health information benefits are predominantly accessible to those with the capacity to access, process, and use it, highlighting potential for health technologies to widen but not narrow health disparities.

**Figure 1.**
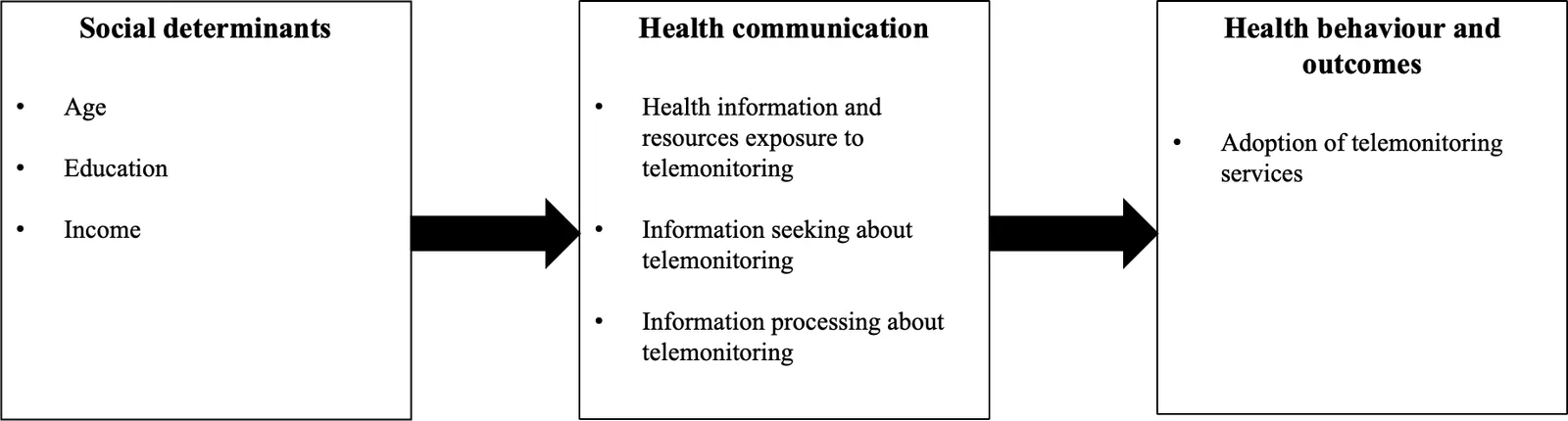
Modified structural influence model in telemonitoring services.

When applied to older adults’ engagement with telemonitoring technologies, the model complements the political economy of aging theory [12,13], which emphasizes how socioeconomic structures create and maintain inequalities in later life. Thus, as shown in Figure 1, we adapted Viswanath et al’s [11] 4-component framework into a 3-component framework centered on socioeconomic determinants (age, education, and income) and their relationship to health information exposure, seeking, and processing related to telemonitoring. Consistent with SIM, the digital health care revolution may disproportionately benefit those with greater socioeconomic resources, technological skills, and health literacy [14]. Friemel [15] showed that the digital divide persists among seniors, encompassing not just technology access but also differences in usage patterns, skills, and perceived benefits. Research has identified social determinants such as age, income, and education as significant factors influencing telemonitoring adoption [16,17].

Despite existing literature linking social determinants and communication outcomes with digital health adoption, significant gaps persist. Socioeconomic status (SES) complexity necessitates diverse social deprivation measures, tailored to research objectives and cultural contexts. While scholars commonly use income, occupation, and education levels to delineate social standing when exploring health disparities [18], Darin-Mattsson et al [19] suggest that in old age, educational attainment may have a weaker association with health compared to wealth, income, and tenure. These findings underscore the importance of focusing on low-income older adults when examining telemonitoring adoption motivators and barriers. Consequently, guided by SIM, we aimed to address the following research question (RQ):

RQ1: What are the motivators and barriers associated with the adoption of telemonitoring services among low-income older adults, as understood through the lens of information exposure, usage, attention, seeking, and processing?

#### Phase 2: Message Framing Strategies for Telemonitoring Acceptance

Building upon Phase 1 findings, which highlighted the importance of affordability, convenience, and health benefits, as well as the challenges posed by preferences for in-person consultations, perceived limitations of remote monitoring, and diminished information processing abilities and health LOC beliefs. Phase 2 aimed to develop and test effective communication strategies to enhance telemonitoring adoption. While Phase 1 identified the key drivers of adoption, altering the technology itself or its accessibility through subsidies, redesign, or infrastructure is better addressed through policy or engineering, and falls out of the scope of health communication. Instead, Phase 2 was designed on the premise that user awareness of its benefits and the perceived relevance of those benefits are essential prerequisites for adoption, regardless of a service’s objective advantages [20]. That indicates that the critical question is not only whether these benefits exist, but whether they are being communicated in a way that resonates with older adults, as prior research has shown that older adults often fail to recognize the personal relevance of technologies even when aware of their potential benefits [21]. Accordingly, experimental stimuli of 2 modalities held core service information constant across framing conditions, varying only how this information was presented.

#### Episodic-Thematic Framing in Message Effects

Framing holds particular promise as a major message format studied across diverse health contexts. It involves strategically presented information where attention is drawn toward specific details [8]. In the context of older adults’ technology adoption, framing effects can be particularly significant given the experience gap*,* the discrepancy between older adults’ life experiences and new technologies' demands [22]. This gap creates ambiguity about the value and relevance of technologies, making the framing of messages particularly influential in shaping perceptions and attitudes.

Framing theory posits that media messages shape audience perception and interpretation by highlighting particular aspects of an issue [8]. Iyengar [23] proposed an influential distinction between episodic framing and thematic framing. Episodic framing focuses on concrete exemplars, isolating single events or individuals while offering engaging narratives to depict broader issues via unknown and ordinary individuals [23]. Thematic framing focuses on a more societal context while offering statistical evidence. These framing approaches intersect with a sociocognitive perspective on aging, emphasizing how older adults’ perceptions, beliefs, and decision-making are shaped by both individual cognitive processes and social contexts [24]. In the health communication context, episodic messages might describe a specific individual’s experience with an illness, while thematic messages might present population-level statistics or attribute the issue to broader social factors [25,26]. In this study, the distinction was operationalized as follows: episodic messages featured a personal account from a named older adult regarding their use of health screening services, while thematic messages presented national survey statistics and referenced community health infrastructure (Experimental Stimuli in Methods section).

Findings from Phase 1 showed that only a small number of individuals believed they were fully responsible for their personal health, while the majority displayed a sense of health responsibility attributions to external parties such as the government or medical professionals. This divergence aligns with research in health communication indicating that responsibility for health outcomes is often attributed to a combination of individual and social or systemic factors [27]. Specifically, older adults’ sense of health agency often involves negotiating between personal responsibility and reliance on health care systems, particularly as physical vulnerabilities increase with age, consistent with evidence that older adults’ perceptions of aging shape their health and functioning [28]. How health messages frame this balance between individual and systemic responsibility may significantly impact their effectiveness. Iyengar’s [23] framework was originally designed to explain how episodic and thematic frames shape audience attributions of responsibility for social issues (ie, attributing responsibility to the individual vs society or institutions); thus, it offers a theoretically grounded perspective for examining whether older adults holding differing views on health responsibility resonate differently with these frames.

The distinction between episodic and thematic framing may be closely related to *exemplification theory* [29]. This theory distinguishes between exemplar-based information (ie, vivid and specific cases) and base-rate statistical information, elucidating the cognitive mechanisms underlying their differing effects. According to the theory, exemplar-based information exerts a disproportionate influence on judgment because it is cognitively more accessible and more readily perceived as representative of broader group characteristics compared to abstract statistical data [29]. Recent research strongly supports this effect: a meta-analysis found that across various communication contexts, exposure to exemplar-based information consistently produced stronger effects than exposure to base-rate information [30]; these effects were most pronounced regarding “first-order reality judgments,” such as assessments of prevalence or risk [31]. From this perspective, episodic framing can be viewed as a form of exemplar presentation, whereas thematic framing corresponds to the presentation of base-rate information; and this offers a cognitive-mechanism-level complement to explanations regarding the persuasive advantage of episodic framing [32]. These characteristics may make exemplar-based information particularly relevant for some older adults. Age-related declines in information processing capabilities [33], together with a preference for emotionally meaningful and concrete content [34], suggest that older audiences may be especially sensitive to the availability heuristic and representativeness heuristic underlying the exemplar effect. Therefore, we propose the following hypothesis:

H1: Compared to those exposed to thematic framing, older adults exposed to episodic framing report more positive (1) attitudes toward and (2) intention to use telemonitoring services.

#### Mediating Role of Attitude

Based on Ajzen’s [35] Theory of Planned Behavior (TPB), attitude is one of the predictors demonstrating strong and consistent relationships with intention to perform a behavior. This theoretical relationship has particular relevance for older adults given innovation resistance often observed in later life [36]. This resistance isn’t merely about technological difficulty but reflects complex attitudinal factors including concerns about privacy, perceived usefulness, and compatibility with existing routines and values [37]. Understanding how attitudes mediate the relationship between message framing and adoption intentions can therefore provide insights into the psychological mechanisms underlying older adults’ technology decisions. Similarly, studies have found evidence supporting significant relationships between attitudes and behavioral intentions in the context of telehealth acceptance and COVID-19 vaccination [38-40]. Thus, we posited:

H2: Attitude toward using telemonitoring services is positively associated with intention to use telemonitoring services.

H3: The relationship between framing and intention to use is mediated by attitude toward using telemonitoring services.

#### Moderating Role of Video and Photo-Text Modality

The moderating role of modality suggests the delivery method of messages through media platforms significantly influences message reception [41]. Defined modalities, categorized into video and photo-text, are informed by dual-coding theory [42], which posits that visual and verbal information is processed by distinct cognitive systems. For older adults, message modality may have heightened importance due to age-related changes in sensory processing and cognitive function. Charness and Boot [43] note that age-related changes in visual and auditory processing can affect how older adults engage with different media formats. This theoretical framework supports that videos, combining visual with conversational elements, can enhance comprehension and retention, making them particularly effective for presenting eHealth information to older adults [44].

Evidence suggests varying impacts based on the chosen modality. One study [45] found that both gain- and loss-framed photo-text stimuli significantly influenced health behavior intentions, whereas another [46] demonstrated that loss-framed videos increased perceived severity of health risks more effectively than gain-framed videos. de Graaf et al [47] and Occa and Suggs [48] highlighted the stronger impact of video messages on health behavior attitudes compared to photo-text modalities. Therefore, we proposed:

H4: Modality and framing would interact, such that participants assigned to the video condition would have a more positive attitude toward using telemonitoring services compared to participants assigned to the photo-text condition.

#### Moderating Role of External LOC

Wallston et al [49] defined health LOC as the degree to which individuals believe their health is controlled by their own behavior versus external forces. People with an internal LOC believe health status is determined by internal causes such as their own behavior. Meanwhile, those with an external LOC believe that one’s health depends on external causes or powerful others such as health professionals. Health LOC intersects with gerontological theories about control beliefs in later life. The selective optimization with compensation model [50] suggests successful aging involves adjusting control strategies as resources and capacities change. External LOC might represent an adaptive response to increasing health challenges rather than simply passive orientation [51]. Prior research supports a strong association between numerous sociodemographic variables and external LOC, with studies finding that those of older age, lower income, and lower education were more external in terms of health LOC [52,53].

Current literature found individuals with a stronger external health LOC are more likely to find thematic framing messages more appealing [54], as they emphasize societal or group responsibility. Similarly, Ceylan and Hayran [55] verified that LOC moderated the effects of prosocial and self-interested messages in following COVID guidelines, where individuals with higher external LOC were more influenced by prosocial messages. In the context of interactive health websites, those with higher external LOC are responding more strongly to vivid, exemplar-rich message formats [56]. However, other studies have shown contrasting or inconclusive results on the link between episodic or thematic framing and individual and societal attributions [57,58]. Hence, the following question was asked:

RQ2: To what extent does external LOC moderate the relationship between framing and attitude toward using telemonitoring services? The proposed research model and hypotheses are presented in Figure 2.

**Figure 2.**
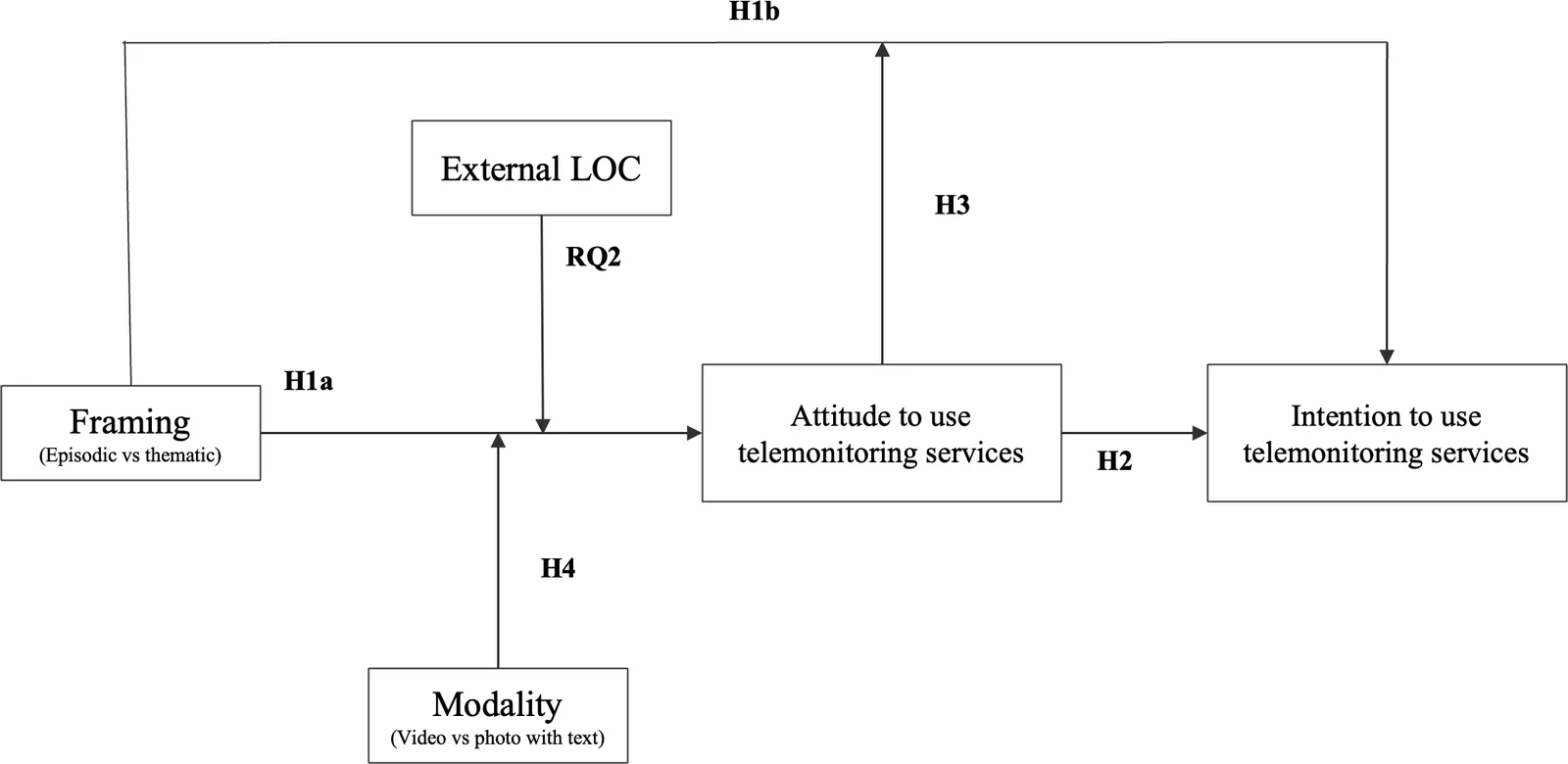
Proposed diagram of the pilot health communication campaign on telemonitoring adoption. H1a: hypothesis 1a; H1b: hypothesis 1b; H2: hypothesis 2; H3: hypothesis 3; H4: hypothesis 4; LOC: locus of control; RQ2: research question 2.

## Methods

### Research Design

This research used a sequential mixed methods design with 2 phases. Phase 1 conducted qualitative interviews and home observations with older adults to identify key motivators and barriers to telemonitoring adoption through the lens of the SIM. Phase 2 experimentally tested how message framing approaches and modalities might influence telemonitoring acceptance among the target population. This sequential design allowed development of evidence-based communication strategies informed by the lived experiences.

### Ethical Considerations

#### Overview

The research was conducted with FullHealth (provided by FullHealth Asia), a grassroots initiative that provides telemonitoring services through health screening and monitoring booths (Figure 3). This study received approval from the Institutional Review Board (IRB) of Nanyang Technological University (IRB-2022‐826). All participants provided written informed consent prior to participation in the study, with protocols designed to accommodate potential age-related needs in the consent process. Before each session, participants were briefed on the study’s goals and their participation rights, with consent for audio-recording obtained and anonymity assured through the use of pseudonyms. All data were deidentified by using numerical codes, and any identifying information mentioned in the interview transcripts was removed. The data were stored securely and were accessible only to research team members (DW, HB, APTQ, GHYH, PSH, ESMO, and EWJL). All participants received S$30 (US $23.40) compensation.

**Figure 3.**
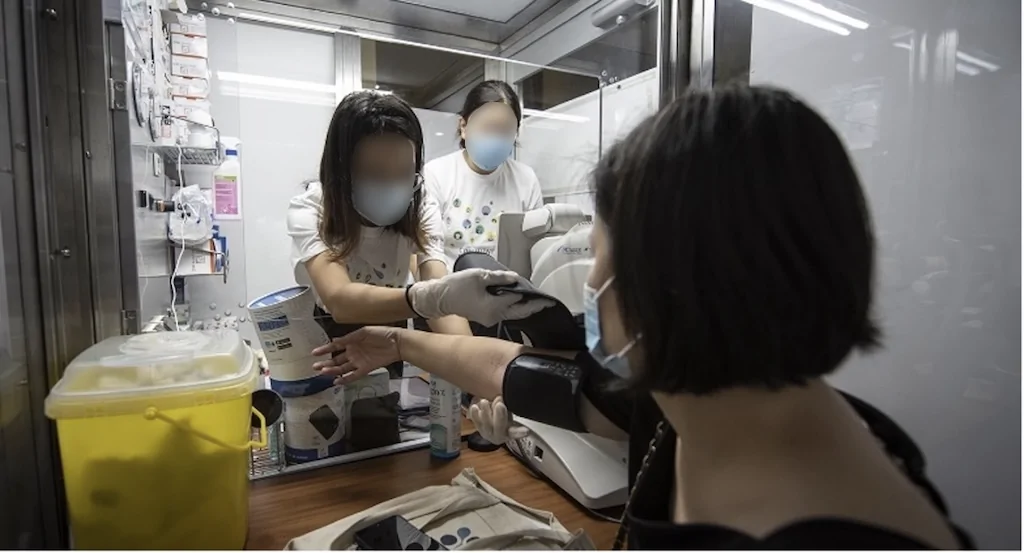
FullHealth telemonitoring booth at community venues.

#### Phase 1: Qualitative Study on Motivators and Barriers

##### Participants and Sampling

Our research focused on older adults in Singapore, a rapidly aging society where telemonitoring services offer significant potential. We targeted Singaporean adults aged 50 years and older to capture current and future older populations. Participants mainly earned ≤S$3000 (US $2340)/person/month, near the national median of S$3287 (US $2563.86) [59], and included English and Mandarin speakers per census data [60]. We recruited participants through convenience sampling facilitated by FullHealth. We included both users and nonusers of the FullHealth telemonitoring service to capture diverse perspectives.

##### Data Collection

Data collection was conducted in person in November 2022, using semistructured interviews and observation guides in English and Mandarin. We conducted pilot sessions with older adults familiar with telemonitoring but not participating in the main study to refine the guides for effectiveness while minimizing cognitive burden. Main sessions, facilitated by trained FullHealth volunteers, lasted approximately 90 minutes each (N=17), with appropriate breaks.

##### Data Analysis

We applied thematic analysis, following Braun and Clarke’s [61] guidelines. The last (EWJL) and corresponding (HB) authors independently coded a subset of the data (4/17, 23.53% of participants) through iterative open coding. After comparing findings, discrepancies were resolved through discussion. The remaining data were then independently coded. Cohen Kappa coefficient (κ=0.74) indicated substantial intercoder agreement [62].

### Phase 2: Experimental Study on Message Framing

#### Study Design

We implemented a 2×2 between-subjects experimental design with framing conditions (episodic and thematic) and media formats (video and photo-text), engaging 120 participants from January 16 to February 1, 2023. Participants were recruited using convenience and snowball sampling through FullHealth Asia, referrals, and social media. All participants were unfamiliar with the FullHealth booth.

#### Experimental Stimuli

*Framing conditions:* thematic framing highlighted societal issues using “Singapore” in titles, statistical data, national surveys, and references to older adult activity centers to reflect broader societal concerns. Episodic framing focused on the individual, using direct address (eg, “You”), personal stories, and specific experiences. Both framing approaches ensured clarity for older audiences.

*Modalities:* Stimuli maintained design consistency through uniform color schemes, fonts, and logos. Photo-text stimuli are provided in Multimedia Appendix 1; the 4 video stimuli are provided in Multimedia Appendices 2–5. Differences were marked by imagery, text, and narration changes for video content, accommodating potential age-related sensory and cognitive changes.

Crucially, the core information of service description, emphasizing that FullHealth’s booth was free, convenient, and offers comprehensive health checks (blood sugar, blood pressure, height, weight, heart, and eyes), remained consistent across the episodic and thematic conditions in both the English and Chinese versions. The only difference between the presentation styles lay in the narrative approach used to convey this information (personal narratives vs aggregate statistics combined with organizational background), isolating the framing manipulation from message content.

#### Procedure

Participants were randomly allocated to 4 conditions: (a) episodic video, (b) episodic photo-text, (c) thematic video, or (d) thematic photo-text. After informed consent, participants assessed external LOC, viewed assigned stimulus, then completed attitude and intention measures plus manipulation checks. A debriefing session clarified any misinformation during the study. Participants received S$15 (US $11.70) as compensation.

#### Measures

External LOC: this moderating variable was measured using a 5-point Likert scale adapted from the Health Locus of Control Scale (HLCS) by Wallston et al [63], using items such as “Good health is largely a matter of good fortune,” with mean 3.13 (SD 0.60, Cronbach α=0.72).

Attitudes toward telemonitoring: participants’ attitudes were measured using a 5-point semantic differential scale, with items modified from Dabholkar and Bagozzi [64]. Participants rated their feelings toward using the FullHealth booth on dimensions such as “Good to bad” and “Harmful to beneficial,” with mean 4.03 (SD 0.47, Cronbach α=0.85).

Intention to use telemonitoring services*:* this was measured on a 5-point Likert scale, adapted from Boontarig et al [65], with items such as “I intend to use this FullHealth booth in the future,” mean 4.14 (SD 0.65, Cronbach α=0.90).

Manipulation check: for framing, participants assessed statements reflecting message focus. Items for episodic framing evaluated FullHealth booth experiences from an individual older adult’s perspective, while thematic framing assessment involved societal impacts and statistical evidence. For modality, 2 items checked participants’ recognition of the stimulus format (video or photo-text).

#### Data Analysis

We analyzed the data using Hayes’ PROCESS macro (Model 9) [66] via SPSS Statistics (IBM Corp), incorporating gender as a covariate. Because PROCESS estimates a series of ordinary least squares regression equations rather than a simultaneously estimated structural equation model, the coefficients reported below reflect independent regression paths but not a jointly estimated path model; accordingly, global model fit indices (such as *χ*², Comparative Fit Index [CFI], and root-mean-square error of approximation [RMSEA]) are not applicable here. Message framing was the independent variable, with attitudes and intentions toward telemonitoring services as dependent variables. External LOC and modality were considered moderating variables. The complete set of model estimation coefficients (including all covariate, interaction paths, and conditional indirect effects at representative values of the moderators) is detailed in Multimedia Appendix 6, together with bootstrap SE and 95% CI.

#### Statistical Power

A sensitivity power analysis indicated that, based on the sample size (N=120) and the 6-predictor moderated regression model used to test Hypotheses 1‐4 and RQ2, the study had 80% power (*α*=.05) to detect an effect of Cohen f²≥.067, an effect size falling between the conventional benchmarks for small (.02) and medium (.15) effects [67]. Post hoc power analyses regarding the key effects reported herein confirm that the study possessed sufficient power to detect the significant “framing× locus of control” interaction (*f²*=.095, power=.92) and the relationship between attitudes and intentions (*f²*=.173, power>.99). The post hoc statistical power for the main effects of framing, presentation modality, and gender, as well as the “framing× presentation modality” interaction, was relatively low (0.06‐0.10) (Table 1).

**Table 1.** Sensitivity and post hoc power analysis results.

Effect	Observed f²	Post hoc power
Framing × External LOC^a^ → Attitude	0.095	0.918
Attitude → Intention	0.173	0.995
External LOC (main) → Attitude	0.018	0.302
Framing × Modality → Attitude	0.004	0.099
Modality (main) → Attitude	0.002	0.082
Framing (main) → Attitude	0.001	0.058

a LOC: locus of control.

## Results

### Phase 1: Motivators and Barriers to Telemonitoring Adoption

The qualitative phase involved 17 participants, 10 telemonitoring users (denoted by “U”) and 7 nonusers (denoted by “N”), aged 56‐86 years. Most participants (15/17, 88.2%) had attained education up to A-level or Diploma, while 11.8% (2/17) had no formal education. The majority (14/17, 82.4%) reported monthly income below S$2000 (Table 2). Analysis revealed 3 key motivators, 3 barriers, and 2 influential factors affecting telemonitoring adoption emerged (Figure 4).

**Table 2.** Demographics of participants in the interview and home observation.

Demographics	Total (N=17)	Users (n=10)	Nonusers (n=7)
Sex, n (%)			
Male	12 (70.59)	9 (90.00)	3 (42.86)
Female	5 (29.41)	1 (10.00)	4 (57.14)
Age (in years), n (%)			
50‐59	4 (23.53)	3 (30.00)	1 (14.29)
60‐69	6 (35.29)	5 (50.00)	1 (14.29)
70‐79	6 (35.29)	1 (10.00)	5 (71.43)
80‐89	1 (5.88)	1 (10.00)	0 (0.00)
Language Spoken, n (%)			
English	4 (23.53)	2 (20.00)	2 (28.57)
Mandarin	12 (70.59)	7 (70.00)	5 (71.43)
English and Chinese	1 (5.88)	1 (10.00)	0 (0.00)
Education, n (%)			
No formal education	2 (11.76)	1 (10.00)	1 (14.29)
Primary 6 or below	5 (29.41)	3 (30.00)	2 (28.57)
Some secondary	3 (17.65)	1 (10.00)	2 (28.57)
N-level or ITE^a^	1 (5.88)	1 (10.00)	0 (0.00)
O-level	4 (23.53)	2 (20.00)	2 (28.57)
A-level or Diploma	2 (11.76)	2 (20.00)	0 (0.00)
Degree	0 (0.00)	0 (0.00)	0 (0.00)
Postgraduate	0 (0.00)	0 (0.00)	0 (0.00)
Monthly Income, n (%)			
Less than S$1,000 (US $780)	6 (35.29)	4 (40.00)	2 (28.57)
S$1,000 to S$1,999 (US $780 to US $1,559.22)	8 (47.06)	6 (60.00)	2 (28.57)
S$2,000 to S$2,999 (US $1,560 to US $2,339.22)	1 (5.88)	0 (0.00)	1 (14.29)
S$3,000 to S$3,999 (US $2,340 to US $3,119.22)	2 (11.76)	0 (0.00)	2 (28.57)

a ITE: Institute of Technical Education.

**Figure 4.**
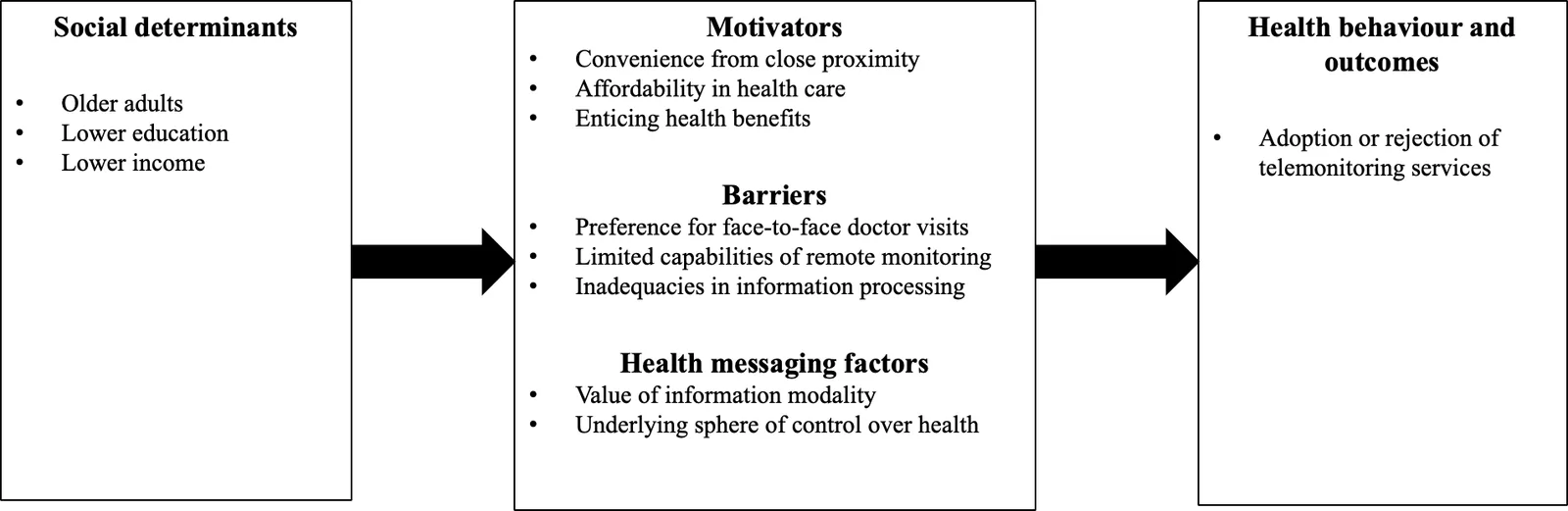
Results of motivators and barriers to telemonitoring services adoption.

### Key Motivators

#### Convenience From Close Proximity

Most FullHealth users cited proximity as the primary reason for using the telemonitoring booth, allowing them to “*save time*” (U7, male, 57 years) and:

*[not] have to travel all the way to [the] polyclinic*.

[U8, male, 63 years]

Nonusers also acknowledged “*convenience*” as a potential motivator. Given the observed mobility challenges, convenience appeared to be particularly significant, reflecting how aging-related changes shape technology preferences.

#### Affordability in Health Care

The “*FOC [Free-of-Charge]*” (U10, Female, 64 years) nature of the booth services encouraged usage among participants. While some noted that the cost advantage might be less significant compared to other subsidized older adult care services (eg, “*This doctor I visit helps me save money. It’s not very expensive because I'm old”* [N1, female, 75 years]), comparing the booth with a regular doctors’ visit, cost remained critical. When discussing more expensive health services, participants expressed concerns:

*Of course, it’s money problems. Every time also doesn’t allow you to cut [medical fees], sum [up] to 37.50 dollars*.

[N7, male, 63 years]

Overall, participants demonstrated high price sensitivity, highlighting how financial considerations become increasingly significant in postretirement life.

#### Enticing Health Benefits

Many participants cited health benefits as a key motivator. These benefits included addressing specific health issues (eg, “*it helps prevent me from getting a stroke again”* [U3, male, 59 years]) and general health monitoring (eg, “*if we have any illness, we will know”* [U8, male, 63 years]). As one participant summarized:

*In conclusion, [FullHealth Booth] is good for our health.*

[U5, male, 86 years]

Participants noted their increased susceptibility to illnesses and, making health monitoring particularly valuable, reflecting how health vigilance increases with advancing age.

### Key Barriers

#### Preference for Face-to-Face Doctor Visits

A primary barrier was the preference for in-person medical consultations. Participants felt that face-to-face meetings provided better understanding of their health concerns (eg, “*Seeing the doctor. Talk about our illnesses [...] clearer. We can understand”* [U4, male, 76 years]; “*When in front of a doctor, if we have any questions, we can ask, which is better”* [U2, male, 57 years]), and offered a sense of familiarity (eg, “*The doctor if we see often already, [we] will go find that doctor”* [N2, female, 78 years]). These preferences highlight how technology adoption in later life is influenced by established relational patterns and trust.

#### Limited Capabilities of Remote Monitoring

The booth’s restricted monitoring capabilities were considered “*very simple*” (U9, male, 65 years) and “*not comprehensive, it’s not full*” (N3, female, 56 years), presenting a barrier to adoption. One participant noted:

*if you go to poly[clinic], one blood test can cross-check a lot of things*.

[N3, female, 56 years]

This perception of limited functionality compared to traditional health care services influenced participants’ willingness to adopt telemonitoring.

#### Inadequacies in Information Processing

Information processing posed a major barrier, especially for lower socioeconomic participants who struggled to understand health advertisements (eg,*“[When shown a health advertisement on the National Steps Challenge] also can't really understand when looking”* [N2, female, 78 years]); *“[When asked what the National Steps Challenge Advertisement is talking about] Don’t know*” (U8, male, 63 years). Information processing abilities varied notably based on education and income levels. Participant U4 (male, 76 years) with no formal education and earned less than S$1000 per month, demonstrated poor information processing skills (*“[When asked about the TraceTogether Advertisement] never studied, don’t understand”* [U4, male, 76 years]). In contrast, participant N3 (female, 56 years), with an O-level certificate and monthly income between S$3000 and S$3999, showed better comprehension (*“[When asked about the TraceTogether Advertisement] To track people where they, in case of Covid, they know who is where, you know, Yeah, so they can trace the people and then inform them that, you know”* [N3, female, 56 years]). These variations highlight how lifelong socioeconomic advantages and disadvantages accumulate to influence technology engagement.

### Factors Influencing Information Processing and Telemonitoring Adoption

#### Value of Information Modality

Participants cited various media sources including “*posters,” “pamphlets,” “flyers,” “radio,” “TV,” “newspapers,” “Facebook,” “WhatsApp,” “YouTube,” “Websites,” and “[digital display screens in] lift.”* Although television and mobile phones were the most commonly owned, neither was frequently used by most participants. Multiple media sources were often mentioned (eg, “*I think from the website, [...] flyers, [...] more news”* [N3, female, 56 years]; *“See the news. [...] I buy newspapers [...] Yes, all [Whatsapp and Facebook] got used, also use Youtube”* [U2, male, 57 years]). The diverse media preferences suggested the importance of considering various modalities in health communication.

#### Underlying Sphere of Control Over Health

Participants displayed varying beliefs about control. Some demonstrated a sense of personal responsibility (eg, *“This [health screenings] is [my] responsibility. If you don’t go, then you have a lot of illnesses, then blame who?”* [U5, male, 86 years]); *“For my blood pressure, if it’s high, I have to control [it]. If my blood sugar is high, my food intake has to [be] control[led]. I think it’s very useful for elderly, people like us to take care of our health*” (U7, male, 57 years). However, most participants showed a tendency to be influenced by health care providers and government authorities (eg, *“[When asked if the participant wanted to undergo a cataract surgery] I then refute back and say you’re a doctor, you then know what, I’m not a doctor how should I know*” [N4, female, 73 years]; *“over there (i.e Chinese medicine clinic) is the volunteer that comes up and helps me do (pump pressure*)” [U3, male, 59 years]). These varying health LOC orientations suggested potential for further investigation (Figure 4).

### Phase 2: Effects of Message Framing on Telemonitoring Acceptance

Building on Phase 1 findings, Phase 2 experimentally tested the effects of different message framing approaches and modalities on telemonitoring acceptance. Participants included 79 (65.8%) women and 41 (34.2%) men, with 98 (81.7%) participants earning below S$3000 monthly. Detailed demographics are presented in Table 3.

**Table 3.** Demographics of participants in the pilot experimental study.

Demographics	Participants (N=120), n (%)
Sex	
Male	41 (34.2)
Female	79 (65.8)
Citizenship status	
Singaporean	117 (97.5)
Permanent resident	3 (2.5)
Age (in years)	
50‐57	19 (15.8)
58‐65	41 (34.2)
Older than 65	60 (50)
Education	
No formal education	1 (0.8)
Primary 6 or below	6 (5)
Secondary	21 (17.5)
N-level or ITE^a^	1 (0.8)
O-level	20 (16.7)
A-level or Diploma	41 (34.2)
Degree	19 (15.8)
Postgraduate	11 (9.2)
Monthly Income	
Less than S$1,000 (US $780)	45 (37.5)
S$1,000 to S$1,999 (US $780 to US $1,559.22)	38 (31.7)
S$2,000 to S$2,999 (US $1,560 to US $2,339.22)	15 (12.5)
Above S$3,000 (US $2,340)	22 (18.3)
Occupation	
Employed full-time	13 (10.8)
Employed part-time	25 (20.8)
Self-employed	13 (10.8)
Not working	12 (10)
Retired	46 (38.3)
Homemaker	11 (9.2)
Housing type	
Rental block	2 (1.7)
Studio apartment	6 (5)
1-room HDB^b^ flat	0 (0)
2-room HDB flat	9 (7.5)
3-room HDB flat	24 (20)
4-room HDB flat	23 (19.2)
5-room HDB flat	30 (25)
Executive condominium	4 (3.3)
Condominium	15 (12.5)
Bungalow or detached house	3 (2.5)
Others	4 (3.3)
Marital status	
Married	77 (64.2)
Single	29 (24.2)
Separated	2 (1.7)
Divorced	6 (5)
Widowed	6 (5)

a ITE: Institute of Technical Education.

b HDB: Housing and Development Board.

### Manipulation Check

*T* test analysis for framing manipulation revealed significant differences between episodic (mean 3.67, SD 0.58) and thematic (mean 2.11, SD 0.55) conditions (*t*_118_= 15.10; *P*<.001). Similarly, the modality manipulation was successful, with participants correctly identifying their assigned condition, with video: mean 4.11, (SD 0.62) versus photo-text: mean 1.80 (SD 0.53, *t*_118_= 22.13; *P*<.001).

### Effects of Message Framing on Attitudes and Intentions

Analysis revealed that framing did not exert a significant main effect on attitudes toward telemonitoring services (b=−.03, *t*_113_=−0.26; *P*=.80). Consistent with this conditional pattern, the direct effect of framing on intentions to use telemonitoring services also did not reach statistical significance (b=−.01, *t*_116_=−0.11; *P*=.91). Participants exposed to episodic framing reported attitudes (mean 4.04, SD 0.52) and intentions to use telemonitoring services (mean 4.14, SD 0.61) broadly similar to those exposed to thematic framing (mean_attitudes_ 4.02, SD_attitudes_ 0.41; mean_intentions_ 4.13, SD_intentions_ 0.58). H1 was therefore not supported as an unconditional hypothesis.

### Relationship Between Attitudes and Intentions

We found a significant positive relationship between attitudes toward using telemonitoring services and intentions to use these services (b=.56, *t*_116_=4.47; *P*<.001), supporting the hypothesis that favorable attitudes positively influence usage intentions (H2). Analysis revealed the unconditional indirect effect of message framing on intention through attitude was not significant, regardless of modality (95% bootstrap CI included zero in both cases). However, this indirect effect was conditional on external LOC (Index=−.28, 95% CI −0.52 to −0.08), while the index for modality was not (Index=.06, 95% CI −0.13 to 0.24). At representative levels of the moderators, framing exerted a significant indirect effect via attitude among participants with low external LOC exposed to video (b=.21, 95% CI 0.02-0.44) and among those with high external LOC exposed to photo-text (b=−.18, 95% CI −0.36 to −0.03). H3 was therefore supported only conditionally, through external LOC.

### Moderating Effects of Message Modality

Contrary to expectations, the analysis showed no significant interaction effect between modality (video vs photo-text) and message framing on attitudes toward telemonitoring services (b=.11, *t*_113_=0.63; *P*=.53). This suggests the modality did not significantly influence how framing affected attitudes in our experimental conditions, indicating that older adults may process different media formats similarly when evaluating health technology information.

### Moderating Effects of External LOC

External LOC significantly moderated the effect of message framing on attitudes toward using telemonitoring services (b=−.49, *t*_113_=−3.28; *P*=.001). When exposed to episodic framing, participants with lower external LOC showed more positive attitudes (mean 4.21, SD 0.47) compared to those with higher external LOC (mean 3.99, SD 0.47). Conversely, thematic framing resulted in more positive attitudes among participants with higher external LOC (mean 4.15, SD 0.47) compared to those with lower external LOC (mean 3.98, SD 0.47). This finding suggests that individuals’ beliefs about health outcome sources play a crucial role in determining the effectiveness of different message framing approaches, reflecting how psychological orientations influence technology adoption decisions in later life.

Figure 5 provides a conceptual summary of the regression paths estimated using Hayes' PROCESS macro (Model 9). The coefficients shown are unstandardized (b) and are taken from the corresponding ordinary least squares regression equations; as noted in the Data Analysis section, global model fit statistics are not applicable.

**Figure 5.**
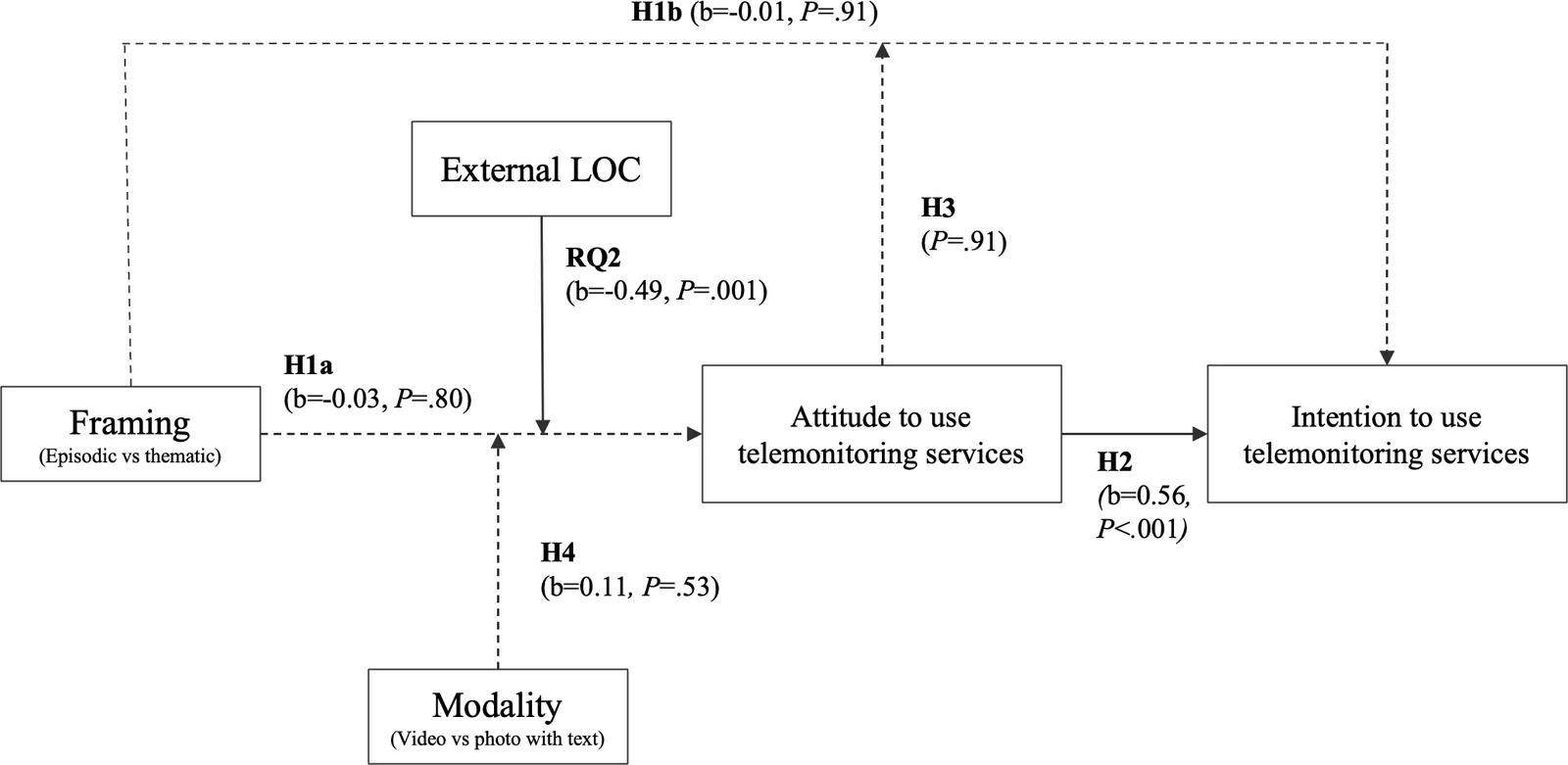
Conceptual summary of regression paths estimated using PROCESS Model 9. H1a: hypothesis 1a; H1b: hypothesis 1b; H2: hypothesis 2; H3: hypothesis 3; H4: hypothesis 4; LOC: locus of control; RQ2: research question 2.

## Discussion

### Principal Findings

Our sequential mixed methods study provides valuable insights into factors influencing telemonitoring adoption among socioeconomically disadvantaged older adults in Singapore. By first identifying key motivators and barriers through qualitative methods then experimentally testing communication strategies, we developed a comprehensive understanding of how to effectively promote telemonitoring acceptance. Our qualitative findings, anchored in the SIM, identified 3 key facilitators of telemonitoring adoption: convenience, affordability, and health benefits. These align with prior research [68,69] and underscore how their absence magnifies existing barriers for older adults with lower socioeconomic status.

Affordability and convenience emerged as essential elements, reflecting the structural theories of aging, wherein socioeconomic constraints often intensify in later life [13]. Prohibitive costs and inconvenience of traditional health care services disproportionately affect those with constrained retirement incomes and limited mobility [70], underscoring how economic inequalities accumulated throughout the life course can restrict technology access and adoption [71]. Perceived health benefits of continuous monitoring served as a powerful motivator, suggesting older adults strategically invest in technologies that help manage age-related health changes and maintain independence [72].

The barriers we identified (preference for in-person consultations, perceived limitations of remote monitoring, and diminished information processing abilities) all relate to SIM components while reflecting age-specific considerations. The preference for face-to-face interactions exemplifies embodied knowledge in health care, showing how accumulated physical and relational experiences shape how older adults interact with health care systems [73]. This reflects established patterns of trust and communication developed throughout the life course [74]. The perceived limitations of telemonitoring relate to information exposure and attention, suggesting a need for clearer communication about the proactive monitoring benefits [1]. Perhaps most critically, the challenge of information processing, exacerbated by cognitive changes and socioeconomic factors [75,76], directly relates to the SIM component of information processing.

### Message Framing, Modality, and Health LOC in Later Life Technology Decisions

Building on our qualitative findings, our experimental study revealed important insights about effective communication strategies for promoting telemonitoring adoption among older adults. Most notably, episodic framing’s persuasive power was conditional on external LOC: our results showed that individuals with lower external LOC responded more favorably to episodic framing, while those with higher external LOC showed greater receptivity to thematic framing. This message-audience matching pattern aligns with a growing body of evidence that dispositional beliefs about control shape which type of health message works [26,55], and a recent systematic review confirms that health LOC systematically conditions how audiences respond to behavioral health interventions more broadly [77]. Substantively, individuals lower in external LOC likely process episodic narratives as personally self-relevant and congruent with their belief that health outcomes are within their own control; individuals higher in external LOC, who attribute health outcomes more to institutions, systems, or chance, may instead find thematic framing more credible and applicable to their own situation, which explicitly foregrounds statistical evidence and institutional infrastructure [34,78,79]. The prevalence of external health LOC aligns with life-span developmental perspectives suggesting that control beliefs often shift toward greater externality with advancing age, reflecting adaptive responses to increasing dependence on health care systems [51]. Older adults’ well-documented preference for emotionally meaningful, narrative information [33] appears to hold most strongly among those who already see themselves as agents in their own health. However, the nuanced interaction between external LOC levels and message framing calls for further exploration, especially to decipher the effects of different dimensions of LOC, such as chance and powerful others, on health communication strategies in diverse aging populations [80].

Contrary to expectations, we found no significant difference in the effectiveness of video versus photo-text modalities in enhancing framing effects on telemonitoring attitudes. This finding diverges from prior research advocating for video’s superior persuasive capacity in health communication [47,48] and challenges simplistic notions about age-related preferences for specific media formats. The lack of observed modality impact suggests that individual differences, such as lifelong media habits, might dilute anticipated modality effects [81,82]. Consequently, the choice of modality for telemonitoring communications should be informed by considerations of content complexity and the cognitive and literacy levels of the target audience [83] while recognizing the diversity of media competencies among older adults.

Taken together, these 2 patterns suggest that what is communicated to older adults, and to whom, matters considerably more than the channel through which it is delivered. Through this lens, external LOC may be more than an individual psychological trait: external LOC may reflect in part how individuals perceive influences beyond their personal agency, from message framing to the behavior of trusted others within one’s social network. Though in this study, the adapted SIM mainly focused on socioeconomic determinants, social capital mechanisms may operate alongside the socioeconomic pathway examined here. Individuals with higher external LOC may be more attuned to influences beyond their own agency when forming health-related judgments. This interpretation resonates with social capital and network-based perspectives in technology adoption research [84,85].

### Digital Inclusion and Aging in Place: Implications for Policy and Practice

Our findings have important implications for inclusive digital health initiatives supporting older adults’ aging in place. First, telemonitoring services should prioritize accessibility and affordability, particularly for socioeconomically disadvantaged older adults through free or subsidized community-based services, within walking distance could enhance adoption, aligning with age-friendly community principles [86]. Singapore’s mature estates could serve as ideal locations for community-based services. Second, communications should position telemonitoring as complementing rather than replacing in-person care, emphasizing continuous monitoring benefits [16] while respecting established health care relationships. Messages could highlight how it complements and enhances doctor-patient relationships by providing continuous data that makes consultations more effective [87]. Third, the effectiveness of episodic versus thematic framing depends on external LOC. Practical guidance should center on message–audience matching rather than promoting a single “best” frame. Episodic narratives are likely to resonate more with older adults lower in external LOC, who tend to see health outcomes as within their own agency, while thematic messages emphasizing institutional involvement and statistical evidence may better suit those higher in external LOC. Communicators should therefore tailor framing to audience segment rather than defaulting to one universal approach, using simple language and visual aids to enhance accessibility. Finally, older adults should be viewed as active cocreators rather than passive recipients [88]. Their concerns reflect rational evaluations based on lifelong experiences. Involving them in design and implementation could enhance both solutions and acceptance.

### Limitations and Future Research

While our study provides valuable insights, several limitations should be noted. First, this study adopted SIM adaptation, which did not incorporate the model’s network-level mediating conditions, such as social capital and peer adoption behavior perception. Future research should directly examine whether social network characteristics or social capital account for this pattern, and integrate validated social network measures (eg, network size, tie strength, and peer adoption status). Second, our qualitative sample size was modest, and the convenience sampling approach may limit generalizability. Future research should expand to include more ethnically diverse participants and explore additional framing approaches. Third, our measure of external LOC would benefit from greater dimensionality in future studies. Longitudinal research examining actual telemonitoring adoption behavior would provide valuable insights. Furthermore, statistical power analysis indicates that the study lacked sufficient power to detect small effects (*f²*<.07); therefore, the nonsignificant main effects of presentation modality and gender should be interpreted with caution and not viewed as definitive evidence of the absence of an association.

### Conclusion

Our research advances understanding of how socioeconomic factors and communication strategies shape telemonitoring adoption among older adults in Singapore. The identified barriers and facilitators illuminate the interplay between aging experiences, health beliefs, and structural inequalities, rather than simple technological resistance. The message–audience matching pattern that episodic framing benefiting participants lower in external LOC, and thematic framing benefiting those higher in external LOC highlights the importance of psychologically tailored communication approaches that recognize older adults’ diverse perspectives. This work contributes to both social gerontology and health communication by demonstrating how targeted interventions can bridge digital divides in aging societies. As Singapore and similar nations develop technological solutions to support aging in place, ensuring these innovations enhance rather than diminish equity, autonomy, and well-being for diverse older populations remains paramount, ultimately helping realize the goal of inclusive, technology-supported aging for all.

## Supplementary material

10.2196/89817 Multimedia Appendix 1 Stimuli posters.

10.2196/89817 Multimedia Appendix 2 Video stimuli: episodic × video experimental stimuli (English).

10.2196/89817 Multimedia Appendix 3 Video stimuli: episodic × video experimental stimuli (Chinese).

10.2196/89817 Multimedia Appendix 4 Video stimuli: thematic × video experimental stimuli (English).

10.2196/89817 Multimedia Appendix 5 Video stimuli: thematic × video experimental stimuli (Chinese).

10.2196/89817 Multimedia Appendix 6 Full Estimated Model (Hayes' PROCESS Macro, Model 9).

## Abbreviations

CFI: comparative fit index
HLCS: Health Locus of Control Scale
IRB: Institutional Review Board
LOC: locus of control
RMSEA: root-mean-square error of approximation
SES: socioeconomic status
SIM: Structural Influence Model
SPSS: Statistical Package for the Social Sciences
TPB: Theory of Planned Behavior
