# Supplementary material for: Telemonitoring Adoption Among Older Adults: Sequential Mixed Methods Study of Message Framing and Structural Influence
Source: JMIR Aging. 2026 Oct 27;9:e89817. doi: 10.2196/89817 (PMC13712255; doi:10.2196/89817)
Supplement: Supplementary file 1 [file aging-v9-e89817-s001.docx]

**Multimedia Appendix 2. Stimuli posters**

**Figure S1.** Pilot campaign episodic x photo-text experimental stimuli (English).

**Figure S2.** Pilot campaign episodic x photo-text experimental stimuli (Chinese).

**Figure S3.** Pilot campaign thematic x photo-text experimental stimuli (English).

**Figure S4.** Pilot campaign thematic x photo-text experimental stimuli (Chinese).
