# Supplementary material for: Telemonitoring Adoption Among Older Adults: Sequential Mixed Methods Study of Message Framing and Structural Influence
Source: JMIR Aging. 2026 Oct 27;9:e89817. doi: 10.2196/89817 (PMC13712255; doi:10.2196/89817)
Supplement: Supplementary file 2 [file aging-v9-e89817-s006.docx]

**Appendix 1. Full Estimated Model (Hayes' PROCESS Macro, Model 9)**

This appendix reports the complete set of estimated coefficients underlying the moderated mediation model presented in the main text, following Hayes' PROCESS macro Model 9 [1,2]. All standard errors and confidence intervals were derived from 5,000 bootstrap resamples. X = message framing (0 = thematic, 1 = episodic); W = external locus of control (mean-centered); Z = modality (0 = photo-text, 1 = video); M = attitude to use TM services; Y = intention to use TM services.

**Table S1.** *Equation 1. Dependent Variable: Attitude to Use TM Services (M)*

*R²* = 0.099, *F*(6, 113) = 2.06, *P* = .063

| **Predictor** | **b** | **t** | ***P*** | **Bootstrap SE** | **95% CI** |
| --- | --- | --- | --- | --- | --- |
| Framing (X) | -0.030 | -0.26 | .798 | 0.104 | [-0.231, 0.173] |
| External LOC (W) | 0.123 | 1.41 | .161 | 0.083 | [-0.042, 0.283] |
| Modality (Z) | 0.061 | 0.52 | .607 | 0.105 | [-0.149, 0.263] |
| Framing × External LOC | -0.494** | -3.28 | <.001 | 0.162 | [-0.811, -0.172] |
| Framing × Modality | 0.105 | 0.63 | .530 | 0.164 | [-0.212, 0.426] |
| Gender (covariate) | -0.018 | -0.20 | .844 | 0.098 | [-0.205, 0.172] |

*Note.* t and p are from the classical OLS coefficient table (as reported in the main text Results); Bootstrap SE and 95% CI are from 5,000-resample bootstrap estimation. The omnibus test for Equation 1 approached but did not reach conventional significance (*P* = .063). This pattern is consistent with a model in which the theorized effect operates through an interaction (Framing × External LOC).

**Table S2.** *Equation 2. Dependent Variable: Intention to Use TM Services (Y)*

*R²* = 0.149, *F*(3, 116) = 6.77, *P* < .001

| **Predictor** | **b** | **t** | ***P*** | **Bootstrap SE** | **95% CI** |
| --- | --- | --- | --- | --- | --- |
| Framing (X, direct effect, c’) | -0.013 | -0.11 | .914 | 0.111 | [-0.234, 0.209] |
| Attitude to Use (M, b-path) | 0.558** | 4.47 | <.001 | 0.134 | [0.314, 0.835] |
| Gender (covariate) | -0.074 | -0.60 | .548 | 0.113 | [-0.291, 0.152] |

*Note.* ** p < .001. The direct effect of framing on intention (c’) was not significant, consistent with full mediation through attitude.

**Table S3.** *Total Effect of Framing on Intention (without Attitude in the model)*

| **Predictor** | **b** | **SE** | **95% CI** | ***P*** |
| --- | --- | --- | --- | --- |
| Framing (X, total effect, c) | 0.002 | 0.128 | [-0.234, 0.254] | .986 |

**Table S4.** *Conditional Indirect Effects: Framing → Attitude → Intention*

| **External LOC** | **Modality** | **Indirect Effect** | **Bootstrap SE** | **95% CI** |
| --- | --- | --- | --- | --- |
| −1 SD | Photo-text | 0.148 | 0.093 | [-0.006, 0.362] |
| −1 SD | Video | 0.207* | 0.107 | [0.018, 0.440] |
| Mean | Photo-text | -0.017 | 0.061 | [-0.125, 0.112] |
| Mean | Video | 0.042 | 0.076 | [-0.106, 0.195] |
| +1 SD | Photo-text | -0.182* | 0.085 | [-0.364, -0.031] |
| +1 SD | Video | -0.124 | 0.096 | [-0.333, 0.048] |

*Note.* * 95% bootstrap CI excludes zero. External LOC values correspond to -1 SD, mean, and +1 SD (mean-centered).

**Table S5.** *Index of Moderated Mediation*

| **Moderator** | **Index** | **Bootstrap SE** | **95% CI** |
| --- | --- | --- | --- |
| External LOC (W) | -0.276 | 0.113 | [-0.522, -0.081] |
| Modality (Z) | 0.059 | 0.093 | [-0.133, 0.238] |

*Note.* The significant index of moderated mediation for External LOC indicates that the strength of the indirect effect of framing on intention (via attitude) varies systematically with participants' external locus of control. The non-significant index for Modality is consistent with the main text's finding that Hypothesis 3 (modality × framing interaction) was not supported.

**References**

1. Hayes AF. Introduction to Mediation, Moderation, and Conditional Process Analysis: A Regression-Based Approach. Guilford Press; 2013.
2. Hayes AF. Introduction to Mediation, Moderation, and Conditional Process Analysis: A Regression-Based Approach. 2nd ed. Guilford Press; 2017.
